# MO Oxygen Therapy Prevents Doxorubicin-Induced Cardiotoxicity

**DOI:** 10.1155/crp/2729462

**Published:** 2025-05-08

**Authors:** Lingjun Zhang, Yanmin Liu

**Affiliations:** ^1^Department of Graduate School, Qinghai University, Xining, Qinghai 810016, China; ^2^Department of Cardiovascular Medicine, Qinghai Provincial People's Hospital, Xining, Qinghai 810007, China

**Keywords:** doxorubicin, heart failure, MO oxygen therapy

## Abstract

**Background:** Micro-oxygen therapy can reduce the effects of doxorubicin (DOX) on left ventricular function, cardiac fibrosis, inflammation, and oxidative stress in SD rats. These results suggest the potential of DOX for clinical use.

**Method:** 8-week-old SPF-grade SD male rats were randomly divided into four groups: control group (Ctrl) (*n* = 10), doxorubicin group (DOX) (*n* = 10), doxorubicin + conventional oxygen intervention group (DOX+CO) (*n* = 10), doxorubicin + micropressed oxygen group (DOX+MO)) (*n* = 10). Left ventricular function was assessed by echocardiography 3 weeks after the end of treatment, and histopathological analysis was conducted utilizing Masson and hematoxylin-eosin (HE) staining. The mRNA expression levels of TGF-β1 and Collagen I were quantified by quantitative real-time PCR (qRT-PCR). Additionally, inflammatory markers, including the concentrations of IL-1β, IL-6, and TNF-α, as well as the activities of SOD and GSH-Px, were measured using enzyme-linked immunosorbent assay (ELISA).

**Results:** The DOX + MO group significantly improved the symptoms of heart failure caused by DOX. The specific results are as follows: The EF significantly increased to 78.037 ± 1.283 (63.259 ± 8.855 in the DOX, *p* ≤ 0.0001); the IVSs increased from 0.243 ± 0.036 to 0.324 ± 0.038 (*p* ≤ 0.001); the LVPWs increased from 0.263 ± 0.028 to 0.323 ± 0.036 (*p* ≤ 0.01); the IVSd and the LVPWd increased from 0.171 ± 0.019 to 0.2 ± 0.015 (*p* ≤ 0.05) and from 0.181 ± 0.032 to 0.234 ± 0.026 (*p* ≤ 0.01). Among cardiac function indexes, NT-proBNP in DOX + MO group was significantly different from that in DOX group (*p* ≤ 0.0001). Compared with DOX group, the degree of myocardial fibrosis in DOX + MO group was decreased, and qRT-PCR showed that MO oxygen effectively reduced the mRNA expression of TGF-β1 and collagen1 induced by DOX. In terms of inflammatory indicators, TNF-α (*p* ≤ 0.0001), IL-1β (*p* ≤ 0.0001), and IL-6 (*p* ≤ 0.0001) in DOX + MO group were significantly lower than those in DOX group. In terms of oxidative stress, serum levels of SOD and GSH-PX were decreased in the DOX group, and MO oxygen therapy effectively prevented the reduction of these indexes. On the other hand, the experimental results also showed that DOX + MO group was significantly better than DOX + CO group in terms of cardiac function, inflammation, and oxidative stress.

**Conclusion:** Microbaric oxygen therapy can reduce the effects of DOX on left ventricular function, cardiac fibrosis, inflammation, and oxidative stress in SD rats. These results provide support for clinical studies to evaluate the potential of DOX in clinical applications.

## 1. Introduction

Heart failure (HF) is a common cardiovascular disease; its occurrence may be related to a variety of factors, such as myocardial damage, overburden of the heart, inflammation, oxidative stress, and neuroendocrine system disorders [1]. The main causes are left ventricular remodeling, fibrosis, ischemia, or reperfusion injury, leading to loss of heart function. The prevalence of symptomatic HF is rising worldwide with increasing population, aging, and the long-term effects of high blood pressure. Despite the variety of current treatments for HF, which mainly rely on drugs and medical devices, these treatments still have many limitations, and the treatment of HF remains complex and challenging, requiring in-depth research [2]. Oxygen therapy has a synergistic effect with drugs, surgery, cardiac rehabilitation, and other treatment methods, which can comprehensively improve the quality of life and prognosis of HF patients [3].

Current medical treatments (such as ACE inhibitors, beta-adrenergic receptor antagonists, and diuretics), while able to relieve symptoms and improve heart function, have limited efficacy for some patients, especially for some specific types of HF (such as HF with ejection fraction retention, HFpEF). Existing drugs often come with significant side effects, such as low blood pressure, impaired kidney function, and electrolyte imbalances. These side effects can affect patient adherence and limit the dosage of the drug used. At the same time, the current treatment methods mainly focus on symptom management and improvement of heart function, but there is still a lack of effective means to improve the repair and regeneration ability of heart tissue. Pathological changes such as myocardial fibrosis are difficult to reverse with existing treatments.

MO therapy is a type of treatment that interferes with the disease by pressurizing and inhaling oxygen in a MO oxygen chamber. MO oxygen therapy can effectively increase blood oxygen partial pressure and improve myocardial hypoxia [4–8]. HF patients are often accompanied by myocardial ischemia and hypoxia. By enhancing the supply of oxygen, both the energy metabolism and functional capacity of the heart can be substantially improved. Oxygen serves as a critical determinant for myocardial cells to produce energy. A sufficient oxygen supply facilitates efficient aerobic respiration in cardiomyocytes, thereby augmenting the synthesis of adenosine triphosphate (ATP). This enhanced energy production not only sustains the contractile function of the heart and boosts its pumping efficiency but also strengthens the heart's resilience to pressure and workload. Furthermore, adequate oxygenation mitigates hypoxic-induced damage and fosters the repair and regeneration of cardiac tissue. Consequently, optimizing oxygen delivery is essential for preserving cardiac health and enhancing cardiovascular functionality. It can also help reduce oxidative stress. By regulating the production and removal of oxygen free radicals in the body, the oxidative damage of cardiomyocytes is reduced, thus combating myocardial fibrosis and cardiac dysfunction. Myocardial hypoxia is one of the important factors for the deterioration of HF patients. Despite the remarkable clinical effect, the exact mechanism of MO oxygen therapy in heart disease still needs to be further studied and explored. Considering the importance of anti-inflammatory and antioxidant stress in the treatment of HF, this study aimed to investigate the effect of MO oxygen therapy on doxorubicin (DOX)-induced HF in rats and its potential mechanism.

## 2. Materials and Methods

### 2.1. Reagents

DOX was purchased from Shanghai Yuanye Biotechnology Co., LTD. (Shanghai, China). We used the following ELISA kits, all of which were purchased by Jiangsu Enzyme-labeled Biotechnology Co., Ltd. (Jiangsu, China) : Rat N-terminal brain natriuretic peptide (NT-proBNP) kit, rat tumor necrosis factor-α (TNF-α) kit, rat interleukin-1 β (IL-1β) kit, rat superoxide dismutase (SOD) kit, rat glutathione peroxidase (GSH-PX) kit, and rat interleukin-6 (IL-6) kit. These kits are used to detect the levels of relevant biomarkers.

### 2.2. Animals

We used 8-week-old SPF-grade SD male rats (weighing 230±20 grams), which were purchased by Beijing Huafokang Biotechnology Co., Ltd. (Experimental Animal License Number: SYXK (Beijing) 2019-0022). After purchase, the rats were raised at the Qinghai Provincial Institute of Endemic Disease Control (Experimental Animal License Number: SYXK (Qing) 2021-0003) and underwent ethical review by the Ethics Committee of Qinghai Provincial People's Hospital. The breeding conditions are as follows: The temperature is (22±2)°C, the relative humidity is (50±5)%, and the light cycle is 12 hours of light /12 hours of darkness. Rats can drink water freely. We change the water bottles every day, clean the cages and bedding regularly, and ensure good ventilation and sufficient light. All animal experiments meet ARRIVE guidelines and are conducted in accordance with the National Institutes of Health Guidelines for the Care and Use of Laboratory Animals.

### 2.3. DOX-Induced HF Experiments in Rats

Forty male SD rats (8 weeks of age) were divided into 4 groups (10 rats/groups) and received the following treatment: Group 1 (control group (Ctrl)), intraperitoneally injected with the same volume of normal saline as DOX group; Group 2 (DOX), doxorubicin hydrochloride was injected intraperitoneally at a dose of 2.5 mg/kg 3 times a week (1 d, 3 d, and 5 d) for 2 weeks, 6 times in total; in Group 3 (DOX + conventional oxygen intervention group (DOX + CO)), the rats received the same dose of DOX injection as Group 2 and were treated with ordinary oxygen therapy, oxygen pressure was 1 ATA, oxygen concentration was 33%, and oxygen flow was 3 L/min, once a day for 2 h each time, for 3 weeks; and in Group 4 (DOX + micropressed oxygen group (DOX + MO)), the rats received the same dose of DOX injection as Group 2 and were treated with micropressed oxygen therapy with micropressed oxygen pressure of 1.3 ATA and oxygen concentration of 23%–25%, once a day for 2 h each time for 3 weeks. After 3 weeks of conventional or microbaric oxygen therapy, the rats were examined by echocardiography, then anesthetized, and euthanized in a carbon dioxide chamber, and then blood and heart samples were collected, respectively.

### 2.4. Echocardiographic Examinations Were Performed

In this experiment, echocardiographic examination indexes of rats were used as the main outcome indexes. After 3 weeks of experiment, the rats were first weighed, anesthetized by inhaling 1%–1.5% isoflurane O2, and then their chest hair was removed and placed on the operating table controlled by 37°C temperature. A sensor probe equipped with 30 MHz was used (RV-707B; Toronto, Canada) with the VisualSONICS Echocardiography system. Myocardial movements were observed and recorded using Vevo 770 V3.0.0 software (VisualSonics Inc., Toronto, Canada).

Left ventricular end-diastolic volume (EDV), left ventricular systolic and end-diastolic internal diameter (LVIDs and LVIDd), ventricular septal thickness (IVSd and IVSs), and left ventricular posterior wall thickness (LVPWd and LVPWs) were obtained from echocardiographic data, and left ventricular EF was calculated using the following formula:

(1) $$\begin{array}{c} \text{EF}\left(\%\right)=\frac{\text{lVIDd}3-\text{lVIDS}3}{\text{LVIDd}3}\times 100. \end{array}$$

### 2.5. Determination of Serum Levels of NT-proBNP, TNF-α, IL-1β, IL-6, SOD, and GSH-PX

After collecting blood samples from rats, the blood samples were stored at room temperature for 2 h, and then centrifuged at 2000 RPM for 20 min. The supernatant (serum) was collected, and the serum was transferred to a new test tube and stored in a −80°C refrigerator until determination. The levels of NT-proBNP, TNF-α, IL-1β, IL-6, SOD, and GSH-PX in serum were detected by corresponding rat ELISA kit.

### 2.6. Ratio of Heart Weight to Body Weight in Rats

Weigh the rats at the end of the experiment. After the blood samples were collected, the heart organs of the rats were taken and weighed. The ratio of heart weight to body weight was observed and calculated.

### 2.7. Histopathological Examination

The hearts of rats were fixed with 10% formalin and embedded with paraffin wax, and 5-μm sections were prepared for HE and Masson Trichrome staining [9]. After full drying, the slides were observed under Zeiss microscope and images were taken.

### 2.8. Real-Time Fluorescence Quantitative PCR (qRT-PCR) Was Used to Determine mRNA Expression

In order to extract total RNA, mouse heart tissue was homogenized with TrIeasy reagent [10] (Yeasen Biotechnology, Shanghai, China). In this study, we used the HyperScript cDNA synthesis kit from EnzyArtisan (Shanghai, China) for reverse transcription to synthesize cDNA. Subsequently, we used the reagents provided by EnzyArtisan and the primers listed in Table 1 to detect the expression levels of transforming growth factor -β1 (TGF-β1) and Collagen1 mRNA through a two-step real-time quantitative polymerase chain reaction (qRT-PCR) method in Table 1.

### 2.9. Data Analysis

Values are expressed as mean ± standard error values (SEM). SPSS 25 statistical software was used to analyze the data. The data were tested for normality and homogeneity of variance. If the data were normally distributed, one-way analysis of variance (ANOVA) was used for comparison among groups, and the method of least significant difference (LSD) test was used for comparison among groups. If the data were not normally distributed, the Kruskal–Wallis test was used for comparison among groups, and *p* < 0.05 was considered statistically significant. The GraphPad Prism9.0 software was used to plot the analysis results.

## 3. Results

### 3.1. Basic Features

**Figure 1:**
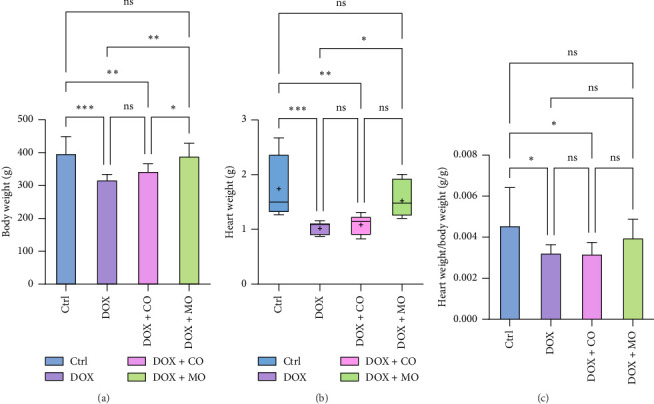
Micropressurized oxygen improves DOX-induced heart failure in rats causing a decrease in body weight, heart weight, and heart weight/body weight ratio. (a) Body weight; (b) heart weight; (c) heart weight/body weight ratio; ^∗^*p* ≤ 0.05, ^∗∗^*p* ≤ 0.01, ^∗∗∗^*p* ≤ 0.001, and ^∗∗∗∗^*p* ≤ 0.0001.

Forty SD rats were divided into the following groups: Ctrl group (*n* = 10), DOX group (*n* = 10), DOX + CO group (*n* = 10), and DOX + MO group (*n* = 10). Nine rats died before the 3-week echocardiographic control (the primary study endpoint), including 3 in the DOX group, 3 in the DOX + CO group, and 3 in the DOX + MO group. There were significant differences in body weight among different experimental groups. In the third week, the comparison of body weight between Ctrl and DOX showed that the body weight of the Ctrl group was 396.74±52.451, while that of the DOX group was 316.886±17.206. This difference was statistically significant (p≤0.001). In addition, the comparison results of body weight between the DOX+MO group and the DOX group also showed significant differences. The body weight of the DOX+MO group was 389.343±39.661, while that of the DOX group was 316.886±17.206. This difference was also statistically significant (p≤0.01). However, the comparison results of body weight between the DOX+CO group and the Ctrl group did not show a significant difference. These results are summarized in Table 2 and Figure 1(a), indicating that the changes in body weight in the DOX group were significantly different from those in other groups, while the DOX+CO group remained relatively consistent with the Ctrl group. Compared with DOX + CO group, the weight loss was more significant in DOX + MO group (389.343 ± 39.661 vs. 342.714 ± 23.686, *p* ≤ 0.05) (Figure 1(a)).

### 3.2. MO Oxygen Improved Left Ventricular Systolic Function in DOX-Induced HF Rats

**Figure 2:**
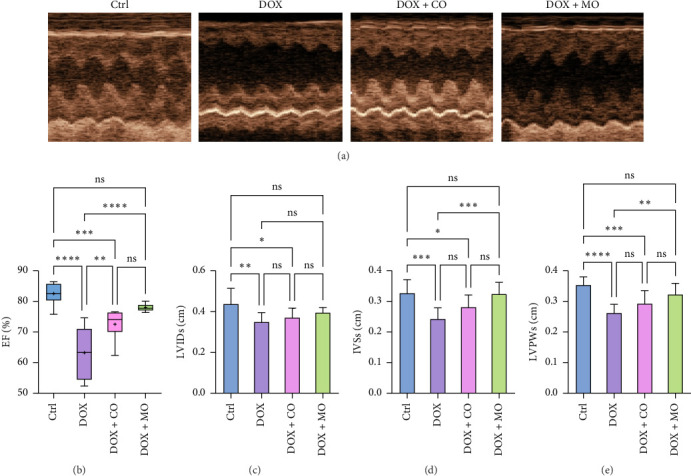
Microcompressed oxygen improves left ventricular function in DOX-induced heart failure rats. (a) Echocardiography performed at the end of 3 weeks of the experiment; (b) EF: ejection fraction; (c) LVIDs: left ventricular internal dimension in systole; (d) IVSs: systolic interventricular septal thickness; (e) LVPWs: systolic left ventricular posterior wall thickness; ^∗^*p* ≤ 0.05, ^∗∗^*p* ≤ 0.01, ^∗∗∗^*p* ≤ 0.001, and ^∗∗∗∗^*p* ≤ 0.0001.

At 3 weeks into the experiment, all groups underwent echocardiographic examinations. Compared to the Ctrl group, the DOX group showed a significant decline in left ventricular function, which was effectively blocked in the DOX + MO group by mild hyperbaric oxygen treatment (Table 2, Figure 2(a)). When compared to the DOX group, the left ventricular systolic function indexes in the DOX + MO group were significantly better. EF was 78.037 ± 1.283 vs. 63.259 ± 8.855 (*p* ≤ 0.0001) (Table 2, Figure 2(b)), IVSs was 0.324 ± 0.038 vs. 0.243 ± 0.036 (*p* ≤ 0.001) (Table 2, Figure 2(d)), and LVPWs was 0.323 ± 0.036 vs. 0.263 ± 0.028 (*p* ≤ 0.01) (Table 2, Figure 2(e)). However, there was no significant difference in LVIDs between the two groups (0.396 ± 0.024 vs. 0.35 ± 0.045, *p* = 0.128) (Table 2). Additionally, there were no significant differences between the DOX + CO group and the DOX group in LVIDs (0.371 ± 0.046 vs. 0.35 ± 0.045, *p* > 0.05), IVSs (0.281 ± 0.039 vs. 0.243 ± 0.036, *p* > 0.05), and LVPWs (0.293 ± 0.042 vs. 0.293 ± 0.042, *p* > 0.05).

### 3.3. MO Oxygen Improved Left Ventricular Diastolic Function in DOX-Induced HF Rats

**Figure 3:**
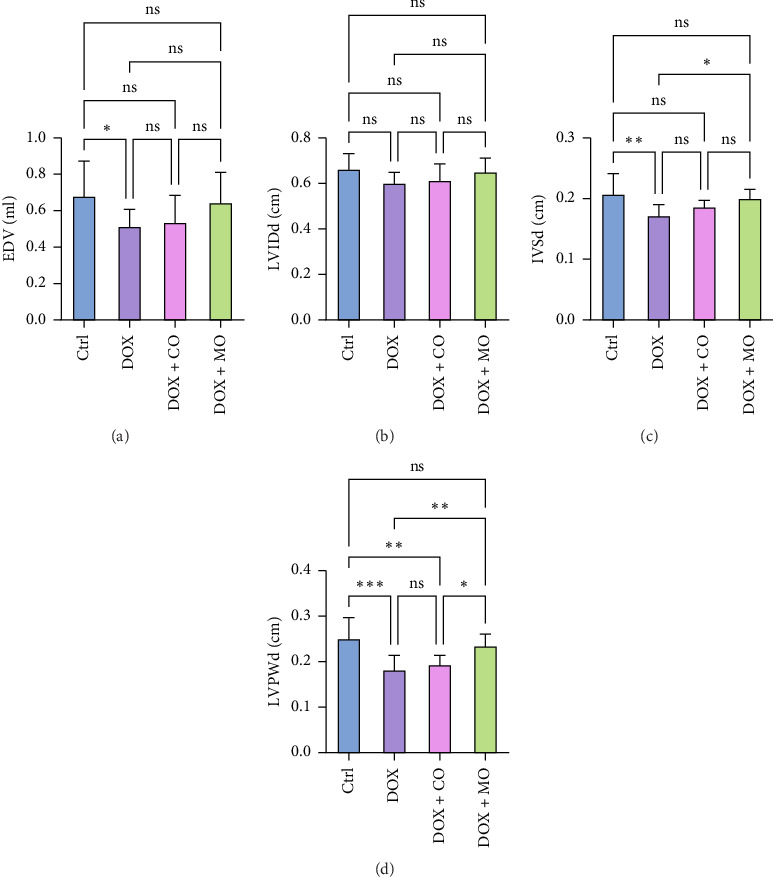
Microcompressed oxygen improves left ventricular function in DOX-induced heart failure rats. (a) EDV: ejection fraction; (b) LVIDd: left ventricular internal dimension-diastole; (c) IVSd: diastolic interventricular septal thickness; (d) LVPWd: diastolic left ventricular posterior wall thickness; ^∗^*p* ≤ 0.05, ^∗∗^*p* ≤ 0.01, ^∗∗∗^*p* ≤ 0.001, and ^∗∗∗∗^*p* ≤ 0.0001.

Compared with the Ctrl group, left ventricular diastolic function indexes EDV, IVSd, and LVPWd decreased significantly in the DOX group (Table 2, Figure 3); however, LVIDd also decreased in the DOX group, but there was no significant difference between the two groups (0.662 ± 0.069 vs. 0.6 ± 0.049, *p* > 0.05). Compared with DOX group, IVSd and LVPWd in DOX + MO group were better, and EDV and LVIDd groups showed no significant difference. IVSd between the two groups showed that DOX + MO was 0.2 ± 0.015, while DOX was 0.171 ± 0.019, and the difference was statistically significant (*p* ≤ 0.05) (Table 2, Figure 3(c)). In addition, there was also a significant difference in the LVPWd between the two groups. It was 0.234 ± 0.026 for DOX + MO and 0.181 ± 0.032 for DOX. The difference was also statistically significant (*p* ≤ 0.01) (Table 2, Figure 3(d)). For ventricular EDV, DOX + MO was 0.643 ± 0.167, while DOX was 0.513 ± 0.095. Although there was a difference, it did not reach statistical significance (*p* > 0.05) (Table 2, Figure 3(a)). Similarly, in the comparison of LVIDd, DOX + MO was 0.65 ± 0.062, and DOX was 0.6 ± 0.049. The difference was also not statistically significant (*p* > 0.05). These results indicate that although some cardiac structure parameters show significant differences among different groups, others do not show significant differences. Between DOX + CO and DOX groups, EDV (0.534 ± 0.150 vs. 0.513 ± 0.095, *p* > 0.05), LVIDd (0.611 ± 0.074 vs. 0.6 ± 0.049, *p* > 0.05), IVSd (0.186 ± 0.011 vs. 0.171 ± 0.019, *p* > 0.05), and LVPWd (0.193 ± 0.021 vs. 0.181 ± 0.032, *p* > 0.05) had no significant difference. LVPWd was significantly better in DOX + MO group than in DOX + CO group, and there was a significant difference between the two groups (0.234 ± 0.026 vs. 0.193 ± 0.021, *p* ≤ 0.05). During the experiment, we observed that compared with the Ctrl group, the rats in the DOX group had loss of appetite, decreased movement, shortness of breath, and ascites. On the contrary, these symptoms were milder in the DOX + CO group and milder in the DOX + MO group than in the DOX group. Compared to the Ctrl group, DOX caused a significant decrease in body weight in the rats, with a notable difference between the two groups (316.886 ± 17.206 vs. 396.74 ± 52.451, *p* ≤ 0.001) (Table 2, Figure 1(a)). In comparison to the DOX group, the DOX + MO group significantly blocked the decrease in body weight (389.343 ± 39.661 vs. 316.886 ± 17.206, *p* ≤ 0.01), showing a significant difference between the two groups.

Compared to the Ctrl group, DOX led to a significant reduction in heart weight, with a notable difference between the two groups (*p* ≤ 0.001) (Table 2, Figure 1(b)). Compared to the DOX group, the DOX + MO group showed a significant improvement in heart weight (1.018 ± 0.124 vs. 1.523 ± 0.326, *p* ≤ 0.05), indicating a significant difference between the two groups. However, regarding heart weight to body weight ratio, no satisfactory results were obtained when comparing DOX and DOX + MO (0.004 ± 0.001 vs. 0.003 ± 0.000, *p* > 0.05) (Table 2, Figures 1(b) and 1(c)).

### 3.4. Micropressurized Oxygen Improved Myocardial Fibrosis in DOX-Induced HF Rats

**Figure 4:**
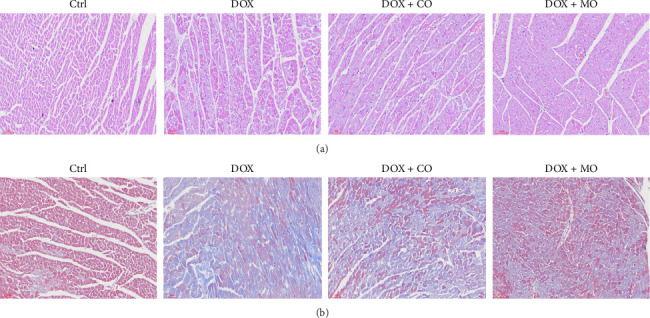
(a) Tissue sections stained with HE. In the Ctrl group, the staining of cardiac tissue was uniform and the myocardial cells were neatly arranged. The myocardial cells in the DOX group were disordered in arrangement and inflammatory cells were more common. The inflammatory cells in the treatment group were significantly reduced compared with those in the DOX group, while the DOX+MO group was superior to the DOX + CO group. (b) Tissue sections stained with Masson Trichrome. In the Ctrl group, the staining of cardiac tissue was in a normal state. However, a large amount of collagen fiber deposition could be observed in the DOX group. Treatment group: compared with the DOX group, the collagen fiber deposition in the treatment group was reduced, and the reduction of collagen fiber deposition in the DOX + MO group was better than that in the DOX + CO group.

**Figure 5:**
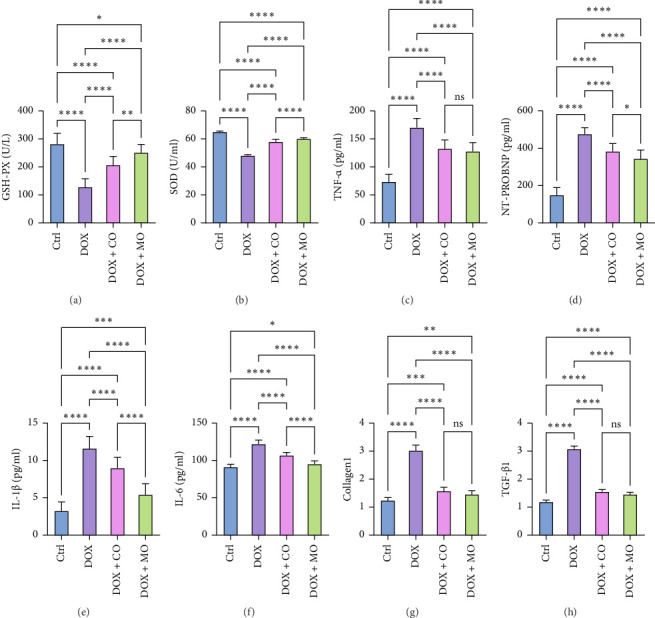
Microcompressed oxygen restores well the reduced expression of antioxidant molecules, blocked inflammatory factor levels with serum markers of myocardial injury, and reduced profibrotic factors. (a) GSH-px: glutathione peroxide oxidizing enzyme; (b) SOD: superoxide dismutase; (c): TNF-α: tumor necrosis factor-α; (d): NT-proBNP: serum markers of myocardial injury; (e) IL-1β: interleukin-1; (f) IL-6: interleukin; (g) collagen1; (h) TGF-β1 : transforming growth factor-β; ^∗^*p* ≤ 0.05, ^∗∗^*p* ≤ 0.01, ^∗∗∗^*p* ≤ 0.001, and ^∗∗∗∗^*p* ≤ 0.0001.

**Figure 6:**
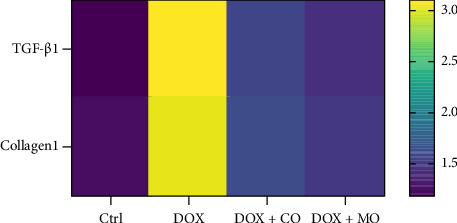
Heatmap, mRNA content of TGF-β1 vs. collagen1 in each group.

HF was induced by intraperitoneal injection of DOX for 2 weeks, and the rats were killed after CO or MO oxygen treatment for 3 weeks. HE staining and Masson Trichrome staining were used to observe the effects of DOX group and treatment group on myocardial fibrosis in cardiac pathological sections. In the tissue sections stained by HE (Figure 4(a)),cardiomyocytes in the Ctrl group were arranged neatly, while myofibrillar opacity and disordered arrangement of cardiomyocytes were observed in rats treated with DOX, and collagen fibers and inflammatory cells were significantly reduced in the tissue sections treated with CO or micropressure oxygen, and the performance of the tissue sections treated with micropressure oxygen was better than that treated with CO. In tissue slices stained by Masson Trichrome (Figure 4(b)), it is obvious that a large amount of collagen fibers have been deposited in DOX tissue slices, and the deposition of collagen fibers has been significantly reduced in the treatment group. Micropressure oxygen effectively blocks this phenomenon. Myocardial fibrosis is a complex pathological process caused by many factors. TGF-β1 is considered to be the central factor promoting fibrosis, and collagen1 is considered to be positively correlated with cardiac failure pathologic progression and mortality. In order to determine the effect of micropressed oxygen on fibrosis, we used qRT-PCR to detect the contents of TGF-β1 and collagen1. It was found that DOX induced mRNA expression of TGF-β1 and collagen1, and micropressed oxygen significantly reduced the expression of these mRNAs (Figures 5(g) and 5(h)). Heat maps (Figure 6) further confirmed the mRNA levels of TGF-β1 and collagen1 in each group. These results suggest that microbaric oxygen significantly alleviates DOX-induced fibrosis in rats by inhibiting the expression of profibrotic molecules and blocking pathological progression.

### 3.5. Micropressurized Oxygen Attenuated Oxidative Stress in DOX-Induced HF Rats by Increasing the Expression of Antioxidant Enzymes

Glutathione (GSH) is the main intracellular antioxidant. SOD is an important antioxidant enzyme that regulates cellular REDOX homeostasis. Compared with Ctrl group, GSH-PX and SOD were significantly reduced in DOX group (Figures 5(a) and 5(b)). Compared with the DOX group, both DOX + CO and DOX + MO groups blocked this decrease well (*p* ≤ 0.0001). However, the elevated level in DOX + MO group was significantly better than that in DOX + CO group (*p* ≤ 0.0001). The final results indicated that MO oxygen therapy could effectively block the DOX-induced decrease in antioxidant enzyme levels.

### 3.6. MO Oxygen Therapy Reduced the Serum Level of NT-proBNP in DOX-Induced HF Rats

NT-proBNP, the precursor of amino-terminal brain natriuretic peptide, is an important serum biomarker of myocardial injury and is used to determine the presence or absence of HF and the severity of the disease in rats. Compared with the Ctrl group, NT-proBNP was significantly increased in the DOX group. Compared with the DOX group, both DOX + CO and DOX + MO groups blocked this increase well (*p* ≤ 0.0001), but the most obvious thing was that micropressure oxygen reduced NT-proBNP better than CO (DOX + MO vs. DOX + CO, 343 ± 46.58 vs. 383 ± 42.17, *p* ≤ 0.05) (Figure 5(d)). The above evidence suggests that micropressurized oxygen can effectively block DOX-induced myocardial injury in order to protect cardiac function.

### 3.7. Microcompressed Oxygen Reduces the Level of Inflammation in DOX-Induced HF Rats

HF with reduced ejection fraction was closely associated with increased levels of proinflammatory factors. The contents of TNF-α, IL-1β, and IL-6 inflammatory factors were determined by ELISA to observe the effect of MO oxygen therapy on inflammation. Compared with the Ctrl group, the contents of TNF-α, IL-1β, and IL-6 in DOX group were significantly increased. Compared with the DOX group, the increase of inflammatory cytokines was well blocked in the treatment group. There were significant differences in TNF-α (*p* ≤ 0.0001), IL-1β (*p* ≤ 0.0001), and IL-6 (*p* ≤ 0.0001) between DOX + MO and DOX + CO groups and DOX group. It should be noted that there were significant differences in IL-1β (*p* ≤ 0.0001) and IL-6 (*p* ≤ 0.0001) reduction between DOX + MO and DOX + CO groups (Figures 5(c)–5(f)). In summary, MO oxygen can effectively reduce the occurrence of HF inflammation, and the effect is significantly better than CO therapy.

## 4. Discussion

HF is caused by a decrease in the heart's pump function, leading to reduced ejection and venous blood stasis [11, 12]. It is often considered the final stage of various cardiovascular diseases. DOX, a common anthracycline [13], plays a significant role in modern cancer treatment and has been included in the World Health Organization's (WHO's) list of essential medicines [12, 13]. However, current studies indicate that the incidence of clinical HF rises significantly during DOX treatment [14]. Our results show that in DOX-induced HF model rats, MO oxygen therapy can improve cardiac dysfunction caused by anthracyclines and reduce myocardial fibrosis, inflammation, oxidative stress, and apoptosis, with this effect achieved by antagonizing the expression of altered molecules.

In 1960, Dutch scholar Boerema published a seminal paper titled “Life without Blood” [15], which garnered significant global attention. This study demonstrated that in a hyperbaric oxygen environment of 3 atmospheres, even with a reduction in hemoglobin content to 0.1 g%, normal blood flow did not occur in the blood vessels of experimental animals. However, the partial pressure of dissolved oxygen reached 21 times that of breathing air under normal pressure, providing ample oxygen for sustaining life. Consequently, these animals were able to maintain a state called “bloodless life” for a limited duration. Hyperbaric oxygen therapy can elevate the partial pressure of oxygen within patients' bodies and enhance myocardial oxygen diffusion capacity, thereby ensuring sufficient oxygen supply to hypoxic cardiomyocytes and improving myocardial contractility as well as cardiac ejection fraction. Additionally, it can facilitate collateral circulation formation at the site of heart infarction and improve myocardial microcirculation to provide adequate oxygenation and blood supply to ischemic cardiomyocytes. Nevertheless, due to limitations related to equipment size, safety concerns, cost factors, and pressure considerations, hyperbaric oxygen technology has not been widely employed in HF treatment. Therefore, is there an alternative treatment modality that can compensate for the shortcomings associated with hyperbaric oxygen therapy? The advent of micropressure chambers has made this treatment approach more accessible and applicable.

Oxygen is the fundamental component of cellular aerobic metabolism, as it enters the respiratory pathway and blood vessels through convection, diffuses across the capillary wall to reach the interstitium, and ultimately reaches the mitochondria where it facilitates cellular respiration [16]. Oxygen is indispensable for all aerobic organisms. Normal oxygenation refers to the optimal oxygen levels required for normal physiological processes. Recently, studies have demonstrated that HBOT induces an increase in reactive oxygen species (ROS) with a kinetic profile similar to that of plasma ROS at 1.4 and 2.5 ATA. Peak production occurs approximately after 2 h and remains elevated above baseline levels for 48 h. Due to their low concentration within mitochondria [17], even minor alterations in oxygen levels can serve as potent triggers for metabolic signals. Mitochondria play a pivotal role in oxygen utilization since cellular respiration takes place therein, with glucose serving as ATP's energy source while oxygen acts as its ultimate electron acceptor in the respiratory chain.

The micropressure oxygen chamber, also known as the micropressure oxygen therapy instrument, utilizes a combination of molecular sieve oxygen production and oxygen pressure technology to achieve dual oxygen supply, ensuring sufficient oxygen delivery to the body's organs. The conventional configuration of the micropressurized oxygen chamber ranges from 1.2 to 1.8 ATA atmospheric pressure, with an ambient oxygen concentration between 23% and 28% [17]. In contrast to hyperbaric chambers operating at pressures of 2 to 3 ATA, this study employs a micropressurized chamber set at 1.3 ATA due to its advantages in terms of portability, safety, and ease of operation. Through the synergistic effect of micropressure and enriched oxygen content, the micropressure oxygen chamber enhances cellular oxygenation by increasing dissolved oxygen concentration and improving blood-oxygen diffusion rate and reserve capacity. Consequently, it rapidly ameliorates myocardial hypoxia and hemodynamic disorders while simultaneously enhancing organ function efficiency through improved tissue perfusion and accelerated metabolism stimulation. Building upon the principles underlying hyperbaric therapy for HF treatment, this study investigates changes in HF indexes before and after MO oxygen therapy apparatus intervention in rat models with HF, thus elucidating MO's efficacy for treating HF in rats while providing empirical support for future clinical applications. Finally, the anti-inflammatory effect of MO has been widely recognized. Studies have shown that it can effectively alleviate the inflammatory response caused by various sports injuries. MO reduces the body's inflammatory response by regulating the inflammatory mediators in the body. In addition, MO has the ability to improve blood flow to the brain and promote the repair and regeneration of brain cells, thereby enhancing the cognitive function and recovery ability of the brain. This dual effect makes MO particularly important in sports medicine.

### 4.1. Effects of Micropressure Oxygen on Left Ventricular Function

The progression of anthracycline-induced HF can be observed from symptomatic HF with clinical signs to an asymptomatic decline in systolic function. Left ventricular ejection fraction (LVEF) is utilized as a quantitative measure for this change, and monitoring fluctuations in EF plays a crucial role in assessing left ventricular function. The primary cardiac effects of anthracyclines involve alterations in cardiac structure, including reduced left ventricular wall thickness, decreased myocardial mass, and diminished ventricular compliance [18, 19]. Our study investigated the measures of systolic and diastolic function of the left ventricle using echocardiography. The findings demonstrated that microbarometric oxygen significantly ameliorated DOX-induced EF as well as left ventricular systolic and diastolic dysfunction, with microbarometric oxygen exhibiting superior efficacy compared to CO therapy in enhancing left ventricular function [20–24].

### 4.2. Effects of Microcompressed Oxygen on Myocardial Fibrosis

Myocardial fibrosis is an important link in the development of HF [18]. The toxic effects of cardiomyocytes are manifested through a variety of molecular mechanisms, primarily by affecting the mitochondrial electron transport chain and the production of ROS by NADPH oxidase (NOX) and nitric oxide synthase. Excess ROS can lead to mitochondrial dysfunction, endoplasmic reticulum stress, calcium release, and DNA damage, eventually leading to dysfunction or cell death of cardiomyocytes [25]. These pathophysiological processes contribute to the atrophy and fibrosis of the heart muscle. In addition, excessive deposition of fibroblasts, which play a key role in cell signaling and myocardial remodeling, promotes poor remodeling of the heart muscle [19]. Cardiac interstitial fibrosis has been reported in both animals and humans treated with DOX and may be associated with oxidative stress. ROS production is a major driver of TGF-β stimulation, and activation of TGF-β triggers activation of linked kinase (ALK). Transcriptional regulation is activated through the phosphorylation of Smad2 and Smad3 by ALK, which subsequently binds to Smad4 and transfers to the nucleus [24, 25]. Therefore, upregulation of TGF-β is closely related to physiological tissue repair and pathological cardiac fibrosis [26]. As a transgenic tool, collagen1 induction can locate the expression of Cre recombinase in cells that actively express collagen, which becomes a clear marker of cardiac fibroblasts in adult tissues and is highly expressed during the development and disease of fibroblasts [19]. Ultrastructural changes in endocardial myocytes induced by anthracyclines included disturbance and loss of myofibril, excessive deposition of fibroblasts, mitochondrial swelling, and cytoplasmic vacuolation [25–28]. Our results showed that microbarometric oxygen therapy significantly improved DOX-induced myocardial myofibrillar deposition in pathologic outcomes and effectively reduced TGF-β1 and collagen1 mRNA levels, which was superior to CO therapy.

### 4.3. Effects of MO Oxygen Therapy on Inflammation

Neurohumoral and sympathetic nervous system activation, oxidative stress, mitochondrial dysfunction, endoplasmic reticulum stress, and calcium treatment play important roles in the onset and progression of HF. In a failing heart, the increased number of damaged mitochondria triggers the accumulation of ROS and apoptosis-associated proteins, along with subcellular inflammation, which ultimately leads to the death of cardiomyocytes and potentially fatal outcomes of HF. In addition, the infiltration of inflammatory cells forms a vicious cycle with neurohumoral activation, further aggravating the damage and death of cardiomyocytes, leading to myocardial fibrosis, and causing cardiac dysfunction and the occurrence of HF [27–31]. Studies have shown that the circulating levels of TNF are significantly elevated in patients with HF [30]. Immunoproteasome, as an effective proteolytic mechanism, is largely expressed in immune cells, but also in nonimmune cells during inflammation. This immune proteasome is induced by interferon-gamma and TNF-α and plays a key role in the degradation of oxidized proteins. Mitochondrial DNA (mtDNA) [31], on the other hand, contains unmethylated CpG sequences, and autophagy escaping mtDNA triggers inflammation and leads to HF. When cardiomyocytes are stressed, cytokines such as IL-1β and IL-6 are produced if DNase II is ablated, resulting in inadequate degradation of mtDNA in autolysosomes [32]. Our study showed that microbaric oxygen therapy significantly prevented the elevation of DOX-induced inflammatory cytokines and reduced the inflammatory response.

In some tissues, repeated micropressure of oxygen can make cells in the body overexpress antioxidant genes [33], resulting in decreased mitochondrial activity and partially reduced ROS production. However, in the long term, antioxidant activity increases, helping mitochondria function without altering the REDOX balance and even increasing their activity [34]. At the same time, the anti-inflammatory effect of the body leads to vasoconstriction, reducing edema and inflammation. It also reduces the production and release of proinflammatory cytokines by neutrophils and monocytes [35]. By reducing the release of proinflammatory factors, such as IL-1, IL-6, TNF-α, and TGF-β1, the damage of inflammatory responses to tissues can be significantly reduced. Therefore, inhibiting the production of these proinflammatory factors not only helps to alleviate the inflammatory response but also protects tissues from damage and promotes repair and regeneration.

### 4.4. Effects of Micropressed Oxygen on Oxidative Stress

Increased oxidative stress is a key mechanism of cardiotoxicity induced by anthracyclines [36–38]. ROS are a natural byproduct of cellular metabolism, and oxidative stress occurs when ROS production exceeds the capacity of the oxidative defense system, or when antioxidant pathways are suppressed. Anthracycline-induced apoptosis of heart cells appears to occur through mitochondrial pathways involving cytochrome C (CytC) and caspase. The production of ROS prompts the release of CytC, suggesting that anthracycline-induced ROS production may contribute to apoptosis through this mechanism [39, 40]. In addition, NOX is also involved in this process, and anthracyclines stimulate NOX activity and further increase ROS production [41], leading to hypertrophy, contractile dysfunction, apoptosis, and fibrosis of cardiomyocytes [42]. Anthracyclines induce mitochondrial toxicity through superoxide anion accumulation and decreased ATP production, which in turn leads to cardiac dysfunction and apoptosis. At the same time, mitochondrial stress and NOX activation lead to the increase of ROS, thus triggering ER stress, which further enhances ROS production, forming a vicious cycle [35, 36]. Cellular oxidative stress induced by anthracyclines may be an early link leading to cardiomyocyte death. In response to anthracyclines, the increase of hydroxyl radicals in the heart can reduce anthracycline-induced cell death through inhibition of SOD [37]. In this study, micropressed oxygen was shown to be effective in preventing the DOX-induced decline in SOD levels [38–40]. GSH-PX is an antioxidant enzyme involved in intracellular REDOX reactions, and its abnormal activity may be associated with the occurrence and development of HF [41–45]. Oxidative stress or elevated levels of ROS in myocardia are important causes of DOX-induced heart damage [45, 46]. Therefore, we measured the expression levels of two antioxidant enzymes, SOD and GSH-PX, in heart tissue. The results showed that DOX reduced the mRNA expression levels of SOD and GSH-PX. However, MO oxygen therapy and CO therapy significantly restored the expression of these molecules. The results showed that MO oxygen therapy was significantly more effective than CO therapy in restoring the expression of these antioxidant enzyme molecules [47–49].

### 4.5. Limitations of the Study

The experimental model of DOX-induced HF in rats at high altitude was constrained by various physical conditions, including low temperature and intensified ultraviolet radiation. However, the most formidable challenge arose from the relatively oxygen-deprived environment. Hypoxic conditions stimulate elevated expression of erythropoietin, vascular endothelial growth factor, and glucose transporter, potentially leading to excessive erythropoiesis and subsequent HF [50–54]. At high altitudes with hypoxia prevailing, the incidence of cardiovascular diseases and their complications significantly increases. Currently, there are limited proven methods for treating patients with HF in clinical practice; hence, standard oxygen therapy is considered an empirical adjunctive treatment whose efficacy has been established [54]. Nevertheless, due to the relative lack of oxygen in plateau patients' environment, CO therapy's effectiveness remains restricted. Consequently, we conducted a comparative experiment between micropressure oxygen therapy and CO therapy to explore a more effective auxiliary treatment method for HF management. The results demonstrated that micropressure oxygen therapy exhibited significant superiority over CO therapy in terms of treatment outcomes. Nonetheless, it should be noted that the findings from this comparative study were substantially influenced by the anoxic plateau environment; thus, further validation studies are warranted in plain areas [55–58].

## 5. Conclusion

In this study, micropressure oxygen therapy antagonized DOX-induced HF in rats by improving cardiac dysfunction, inhibiting myocardial fibrosis, reducing inflammatory markers, and increasing antioxidant enzyme molecular content to improve oxidative stress state. Our studies have shown that DOX therapy leads to significant reductions in heart function and body weight in rats, and DOX + MO can effectively reverse these adverse effects. This finding not only provides new insights into the mechanisms of cardiotoxicity induced by DOX but also provides potential intervention strategies for the prevention and treatment of chemotherapy-related heart damage in future clinical practice. Our findings highlight the potential of hyperbaric oxygen therapy to improve heart function and maintain weight, suggesting its potential use in future clinical studies, especially in the patient population receiving chemotherapy drugs such as DOX.

## Figures and Tables

**Table 1:** Primer sequences for qRT-PCR analysis.

Genetics	Forward	Opposite direction	Amplification length
GAPDH	TGATGGGTGTGAACCACGAG	AGTGATGGCATGGACTGTGG	152
TGF-β1	TGGGATCAGTCCCAAACGTC	AGGTGTTGAGCCCTTTCCAG	102
Collagen1	TTTCCCCCAACCCTGGAAAC	CAGTGGGCAGAAAGGGACTT	287

Abbreviations: Collagen1, type I collagen; GAPDH, glyceraldehyde-3-phosphate dehydrogenase; TGF-β1, transforming growth factor β1.

**Table 2:** The experiment ended at 3 week.

	Ctrl	DOX	DOX + CO	DOX + MO
Body weight (g)	396.74 ± 52.451⁣^∗∗∗^	316.886 ± 17.206	342.714 ± 23.686	389.343 ± 39.661⁣^∗∗^
Heart weight (g)	1.741 ± 0.539⁣^∗∗∗^	1.018 ± 0.124	1.083 ± 0.191	1.523 ± 0.326⁣^∗^
Heart weight/body weight (g/g)	0.005 ± 0.002⁣^∗^	0.003 ± 0.000	0.003 ± 0.001	0.004 ± 0.001
EF (%)	82.546 ± 3.344⁣^∗∗∗∗^	63.259 ± 8.855	72.514 ± 4.980⁣^∗∗^	78.037 ± 1.283⁣^∗∗∗∗^
EDV (mL)	0.678 ± 0.195⁣^∗^	0.513 ± 0.095	0.534 ± 0.150	0.643 ± 0.167
LVIDs (cm)	0.438 ± 0.076⁣^∗∗^	0.35 ± 0.045	0.371 ± 0.046	0.396 ± 0.024
LVIDd (cm)	0.662 ± 0.069	0.6 ± 0.049	0.611 ± 0.074	0.65 ± 0.062
IVSd (cm)	0.207 ± 0.034⁣^∗∗^	0.171 ± 0.019	0.186 ± 0.011	0.2 ± 0.015⁣^∗^
IVSs (cm)	0.327 ± 0.044⁣^∗∗∗^	0.243 ± 0.036	0.281 ± 0.039	0.324 ± 0.038⁣^∗∗∗^
LVPWd (cm)	0.25 ± 0.047⁣^∗∗∗^	0.181 ± 0.032	0.193 ± 0.021	0.234 ± 0.026⁣^∗∗^
LVPWs (cm)	0.35 ± 0.026⁣^∗∗∗∗^	0.263 ± 0.028	0.293 ± 0.042	0.323 ± 0.036⁣^∗∗^
Collagen1	1.23 ± 0.11⁣^∗∗∗∗^	3.01 ± 0.19	1.56 ± 0.14⁣^∗∗∗∗^	1.44 ± 0.13⁣^∗∗∗∗^
TGF-β1	1.17 ± 0.07⁣^∗∗∗∗^	3.06 ± 0.11	1.54 ± 0.07⁣^∗∗∗∗^	1.44 ± 0.08⁣^∗∗∗∗^
GSH-PX (U_L)	280.76 ± 38.71⁣^∗∗∗∗^	127 ± 30.43	206.06 ± 31.44⁣^∗^	250.41 ± 29.85⁣^∗∗∗∗^
SOD (U_ml)	64.89 ± 0.73⁣^∗∗∗∗^	48.02 ± 0.77	57.85 ± 1.78⁣^∗∗∗∗^	60 ± 0.75⁣^∗∗∗∗^
IL-1β (pg_ml)	3.23 ± 1.21⁣^∗∗∗∗^	11.58 ± 1.62	8.95 ± 1.44⁣^∗∗∗∗^	5.38 ± 1.47⁣^∗∗∗∗^
IL-6 (pg_ml)	90.89 ± 3.94⁣^∗∗∗∗^	121.85 ± 5.42	106.61 ± 4.18⁣^∗∗∗∗^	94.89 ± 4.41⁣^∗∗∗∗^
NTproBNP	148.41 ± 40.58⁣^∗∗∗∗^	474.66 ± 35.18	383 ± 42.17⁣^∗∗∗∗^	343 ± 46.58⁣^∗∗∗∗^
TNF-α	73.19 ± 13.52⁣^∗∗∗∗^	170 ± 16.38	132.08 ± 16.17⁣^∗∗∗∗^	127.63 ± 15.77⁣^∗∗∗∗^

*Note:* Values are expressed as mean ± standard deviation. Compared to DOX group, ⁣^∗^*p* ≤ 0.05, ⁣^∗∗^*p* ≤ 0.01, ⁣^∗∗∗^*p* ≤ 0.001, and ⁣^∗∗∗∗^*p* ≤ 0.0001.

## Data Availability

The data used in this study included animal experimental data collected during the experiment, including behavioral observation records, physiological measurements, and molecular biology experimental results. All relevant data will be provided upon request. Data can be obtained by sending an email to the corresponding author stating clearly the type of data requested and the purpose of use. Since this study involved animal experiments, all data are subject to relevant ethical and legal requirements. Data will only be shared to ensure the anonymity and ethical compliance of participating animals. Any data provided will not contain individual identification information of the animal.

## Supporting Information

In Supporting Table A, we present the fundamental data of the experimental group, which primarily encompass the experimental grouping, feeding conditions, intervention measures, and key measurement indicators. In Supporting Table B1, Table B2, and Table B3, we provide the *p* values and 95% confidence intervals for the experimental data.
