# Correction to “MO Oxygen Therapy Prevents Doxorubicin‐Induced Cardiotoxicity”

**DOI:** 10.1155/crp/9874050

**Published:** 2026-02-15

L. Zhang and Y. Liu, “MO Oxygen Therapy Prevents Doxorubicin‐Induced Cardiotoxicity,” *Cardiology Research and Practice* 2025 (2025): 2729462, https://doi.org/10.1155/crp/2729462.

In the article, there was an error in the “**Funding**” section, where the contract acceptance number [NO: 2022‐0301‐ZJC‐0219] was mistakenly provided instead of the official contract number [2022‐ZJ‐756]. The corrected section appears below:

**Funding**

This research was supported by the following grants: (1) Scientific Research Guidance Program of Qinghai Provincial Science and Technology Department (No: 2022‐ZJ‐756). (2) 2022, Qinghai Province “Kunlun Talents High‐End Innovative and Entrepreneurship Talents” Project.

The funding was mainly used for the purchase of experimental materials, animal breeding, and management, including daily diet, environmental maintenance and health monitoring, as well as the maintenance and renewal of experimental equipment. The use of the abovementioned funds ensures that we can carry out basic animal experiments efficiently, guaranteeing the reliability of the experimental results and the ethical compliance of the research.

We apologize for this error.

