# Supplementary material for: MO Oxygen Therapy Prevents Doxorubicin-Induced Cardiotoxicity
Source: Cardiol Res Pract. 2025 May 8;2025:2729462. doi: 10.1155/crp/2729462 (PMC12081151; doi:10.1155/crp/2729462)
Supplement: Supporting Information — Additional supporting information can be found online in the Supporting Information section. [file 2729462.f1.docx]

Supplementary table A:Basic data of experimental group

| Group | Number of Samples | Volume of SD Rats(g) | Feeding conditions | | | Key interventions | Key measurement parameters |
| --- | --- | --- | --- | --- | --- | --- | --- |
|  |  |  | temperature(℃) | humidity(%) | Light/dark cycle(h) |  |  |
| Ctrl | 10 | 230± 20 | 22±2 | 50±5 | 12/12 | Normal saline 2.5mg/kg was injected 3 times a week (1d,3d,5d) for 2 weeks | 1, Basic characteristics: rat body weight, heart weight, heart weight/body weight 2. Cardiac ultrasound indicators: Cardiac systolic function indexes included EF, LVIDs, IVSs and LVPWs The indexes of diastolic function were EDV, LVIDd, IVSd and LVPWd 3. ELISA indicators: GSH-PX, SOD, IL-1, IL-6, NTproBNP, TNF-α 4. PCR indicators: collagen1, TGF-β 5. Cardiac pathological sections: Masson Trichrome and HE staining |
| DOX | 10 | 230± 20 | 22±2 | 50±5 | 12/12 | DOX 2.5mg/kg was administered 3 times a week (1d,3d,5d) for a total of 6 injections for 2 weeks |  |
| DOX+CO | 10 | 230± 20 | 22±2 | 50±5 | 12/12 | After the same injection method as the DOX group, the rats were treated with CO, the oxygen pressure was 1ATA, the oxygen concentration was 33%, and the oxygen flow rate was 3L/min, once a day for 2 hours each time for 3 weeks |  |
| D0X+MO | 10 | 230± 20 | 22±2 | 50±5 | 12/12 | After the same injection method as the DOX group, MO treatment was performed, the microbaric oxygen pressure was 1ATA, and the oxygen concentration was 23-25%, once a day, 2 hours each time for 3 weeks |  |

Supplementary tableB1:P-values and 95% confidence intervals for experimental data

| **Group** | **Body Weight** | | **Heart Weight** | | **Heart Weight/Body Weight** | | **EF** | | **LVIDs** | | **IVSs** | |
| --- | --- | --- | --- | --- | --- | --- | --- | --- | --- | --- | --- | --- |
|  | ***P*** | **95%CI** | ***P*** | **95%CI** | ***P*** | **95%CI** | ***P*** | **95%CI** | ***P*** | **95%CI** | ***P*** | **95%CI** |
| **Ctrl vs DOX** | **0.0002** | (41.26,118.5) | **0.0007** | (0.35,1.09) | **0.033** | (0.0001,0.002) | **<0.0001** | (14.03,24.54) | **0.003** | (0.032,0.143) | **0.0002** | (0.043,0.124) |
| **Ctrl vs DOX+CO** | **0.0078** | (15.43,92.62) | **0.0097** | (0.29,1.02) | **0.028** | (0.0001,0.002) | **0.0006** | (4.77,15.29) | **0.019** | (0.011,0.121) | **0.028** | (0.005,0.085) |
| **Ctrl vs DOX+MO** | 0.697 | (-31.20,45.99) | >0.9999 | (-0.14,0.58) | 0.329 | (-0.0006,0.002) | 0.089 | (-0.748,9.76) | 0.126 | (-0.012,0.097) | 0.891 | (-0.037,0.043) |
| **DOX vs DOX+CO** | 0.216 | (-67.69,16.04) | >0.9999 | (-0.46,0.33) | 0.947 | (-0.001,0.001) | **0.002** | (-14.96,-3.55) | 0.467 | (-0.081,0.038) | 0.081 | (-0.082,0.005) |
| **DOX vs DOX+MO** | **0.0014** | (-114.3,-30.59) | **0.016** | (-0.90,-0.11) | 0.261 | (-0.002,0.0005) | **<0.0001** | (-20.48,-9.07) | 0.127 | (-0.105,0.013) | **0.0007** | (-0.125,-0.037) |
| **DOX+CO vs DOX+MO** | **0.0304** | (-88.49,-4.76) | 0.112 | (-0.04,0.83) | 0.235 | (-0.002,0.0005) | 0.057 | (-11.22,0.17) | 0.411 | (-0.083,0.035) | 0.054 | (-0.086,0.0008) |

Supplementary tableB2:P-values and 95% confidence intervals for experimental data

| **Group** | **LVPWs** | | **EDV** | | **LVIDd** | | **IVSd** | | **LVPWd** | | **GSH-PX** | |
| --- | --- | --- | --- | --- | --- | --- | --- | --- | --- | --- | --- | --- |
|  | ***P*** | **95%CI** | ***P*** | **95%CI** | ***P*** | **95%CI** | ***P*** | **95%CI** | ***P*** | **95%CI** | ***P*** | **95%CI** |
| **Ctrl vs DOX** | **<0.0001** | (0.057, 0.124) | **0.0469** | (0.002,0.327) | 0.063 | (-0.003,0.127) | **0.0045** | (0.012,0.059) | **0.0005** | (0.033,0.103) | **<0.0001** | (-180.3,-126.3) |
| **Ctrl vs DOX+CO** | **0.0008** | (0.027, 0.094) | 0.0811 | (-0.019,0.306) | 0.1254 | (-0.015,0.116) | 0.0748 | (-0.002,0.044) | **0.0025** | (0.0219,0.092) | **<0.0001** | (-149.9,-95.92) |
| **Ctrl vs DOX+MO** | 0.066 | (-0.002,0.064) | 0.6612 | (-0.127,0.197) | 0.7103 | (-0.052,0.077) | 0.5473 | (-0.017,0.03) | 0.3684 | (-0.02,0.051) | **<0.0001** | (-105.6,-51.58) |
| **DOX vs DOX+CO** | 0.1001 | (-0.066,0.006) | 0.8051 | (-0.197,0.155) | 0.7443 | (-0.08,0.059) | 0.2616 | (-0.04,0.011) | 0.5447 | (-0.05,0.027) | **0.0284** | (3.361,57.35) |
| **DOX vs DOX+MO** | **0.002** | (-0.096,-0.023) | 0.1423 | (-0.306,0.046) | 0.1609 | (-0.122,0.021) | **0.0298** | (-0.054,-0.003) | **0.0085** | (-0.091,-0.015) | **<0.0001** | (47.71,101.7) |
| **DOX+CO vs DOX+MO** | 0.101 | (-0.066,0.006) | 0.2176 | (-0.285,0.067) | 0.2758 | (-0.109,0.032) | 0.2616 | (-0.04,0.011) | **0.0347** | (-0.081,-0.003) | **0.0019** | (17.35,71.34) |

| **Group** | **SOD** | | **IL-1β** | | **IL-6** | | **NTproBNP** | | **TNF-α** | | **collagen1** | | **TGF-β1** | |
| --- | --- | --- | --- | --- | --- | --- | --- | --- | --- | --- | --- | --- | --- | --- |
|  | ***P*** | **95%CI** | ***P*** | **95%CI** | ***P*** | **95%CI** | ***P*** | **95%CI** | ***P*** | **95%CI** | ***P*** | **95%CI** | ***P*** | **95%CI** |
| **Ctrl vs DOX** | **<0.0001** | (-17.77,-15.96) | **<0.0001** | (-9.538,-7.154) | **<0.0001** | (-34.68,-27.24) | **<0.0001** | (-360.3,-292.2) | **<0.0001** | (-109.6,-84.05) | **<0.0001** | (-1.931,-1.632) | **<0.0001** | (-1.979,-1.805) |
| **Ctrl vs DOX+CO** | **<0.0001** | (-12.88,-11.06) | **<0.0001** | (-6.913,-4.529) | **<0.0001** | (-19.43,-11.99) | **<0.0001** | (-268.6,-200.6) | **<0.0001** | (-71.65,-46.13) | **0.0001** | (-0.481,-0.182) | **<0.0001** | (-0.454,-0.281) |
| **Ctrl vs DOX+MO** | **<0.0001** | (-10.73,-8.918) | **0.0007** | (-3.344,-0.961) | **0.0357** | (-7.72,-0.27) | **<0.0001** | (-228.6,-160.6) | **<0.0001** | (-67.20,-41.69) | **0.0087** | (-0.372,-0.061) | **<0.0001** | (-0.362,-0.181) |
| **DOX vs DOX+CO** | **<0.0001** | (3.984,5.799) | **<0.0001** | (1.432,3.816) | **<0.0001** | (11.53,18.97) | **<0.0001** | (57.66,125.7) | **<0.0001** | (25.16,50.68) | **<0.0001** | (1.288,1.612） | **<0.0001** | (1.431,1.619) |
| **DOX vs DOX+MO** | **<0.0001** | (6.129,7.945) | **<0.0001** | (5.002,7.386) | **<0.0001** | (23.24,30.68) | **<0.0001** | (97.66,165.7) | **<0.0001** | (29.60,55.12) | **<0.0001** | (1.397,1.734) | **<0.0001** | (1.524,1.720) |
| **DOX+CO vs DOX+MO** | **<0.0001** | (1.238,3.053) | **<0.0001** | (2.378,4.762) | **<0.0001** | (7.987,15.43) | **0.0222** | (5.992,74.01) | 0.4864 | (-8.314,17.20) | 0.1707 | (-0.053,0.284) | 0.0528 | (-0.001,0.195) |

Supplementary tableB3:P-values and 95% confidence intervals for experimental data
